# Diabetic kidney disease: integrating multi-omics insights, artificial intelligence, and novel therapeutics for precision medicine

**DOI:** 10.3389/fgene.2026.1760654

**Published:** 2026-01-20

**Authors:** Tao Li, Kaili Chen, Yiting Sun, Linqi Zhang

**Affiliations:** 1 Kidney Disease Diagnosis and Treatment Center, The First Affiliated Hospital of Henan University of CM, Zhengzhou, Henan, China; 2 Collaborative Innovation Center of Prevention and Treatment of Major Diseases by Chinese and Western Medicine Henan Province, Zhengzhou, Henan, China

**Keywords:** artificial intelligence, diabetic kidney disease, metabolomics, precision medicine, single-cell RNA sequencing

## Abstract

Diabetic kidney disease (DKD) remains a leading cause of global morbidity and mortality. While current therapies like sodium-glucose cotransporter 2 (*SGLT2*) inhibitors and glucagon-like peptide-1 receptor agonists (GLP-1RAs) have improved outcomes, significant challenges persist in early detection and halting progression. This review synthesizes recent transformative advances in DKD research. We highlight how single-cell RNA sequencing (scRNA-seq) and spatial transcriptomics have unveiled unprecedented cellular heterogeneity, delineated pathogenic trajectories like maladaptive tubular cell states, and established immune dysregulation as central to disease progression. Concurrently, metabolomics provides a window into early metabolic disturbances, identifying novel biomarkers that reflect mitochondrial dysfunction and oxidative stress. Furthermore, artificial intelligence (AI) is revolutionizing clinical practice, with deep learning models like DeepDKD demonstrating high accuracy in non-invasive screening using retinal images and enabling refined risk stratification. These multi-omics insights are paralleled by the development of novel therapeutic agents targeting inflammation, fibrosis, and metabolic pathways beyond traditional targets. The integration of high-resolution molecular profiling, AI-driven analytics, and mechanism-based therapeutics is paving the way for a new era of precision nephrology, offering hope for earlier intervention and personalized management strategies for DKD.

## Graphical Abstract

**Figure:**
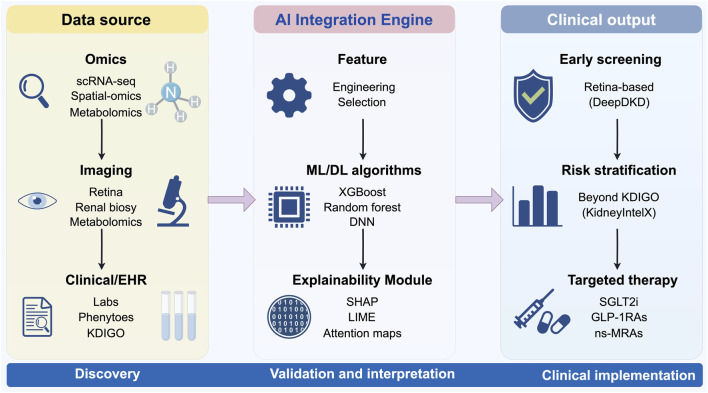
AI-integrated framework for clinical applications in DKD. The figure presents a three-column workflow: Data sources (left, yellow) include omics (scRNA-seq, spatial-omics, metabolomics), imaging (retina, renal biopsy), and clinical/EHR data (labs, phenotypes, KDIGO staging). The AI integration engine (middle, pink) features feature engineering, ML/DL algorithms (XGBoost, random forest, DNN), and explainability tools (SHAP, LIME, attention maps). Clinical outputs (right, blue) progress from early screening (retina-based DeepDKD) to risk stratification (beyond KDIGO using KidneyIntelX) and targeted therapies (SGLT2i, GLP-1RAs, ns-MRAs). The white-background diagram uses color-coded sections and directional arrows to show data flow from inputs through AI processing to clinical implementation.

## 1 Introduction

**FIGURE 1:**
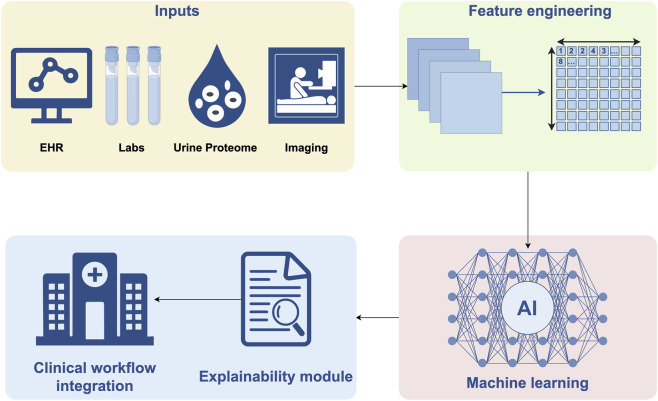
Schematic of an AI-enabled multi-modal diagnostic system for DKD. The conceptual framework illustrates an AI-assisted diagnostic system for improving early detection and risk stratification of DKD. The schematic is organized into four interconnected modules representing sequential steps from data collection to clinical deployment, with a consistent flow from left to right and top to bottom.

DKD represents an increasingly critical global health burden. The management of DKD stands at a pivotal juncture driven by parallel revolutions in high-resolution molecular profiling, AI, and pharmacotherapy (Loftus et al., 2022). However, these groundbreaking advances often progress in silos, creating a critical translational gap between mechanistic discovery and clinical application. There is a pressing need for a synthesized overview that connects insights from multi-omics technologies with the power of AI to decipher molecular complexity and the development of novel therapeutics (Loftus et al., 2022; Balzer et al., 2022; Tan et al., 2024; Pereira et al., 2022). This review is motivated by the urgent necessity to bridge these fields, addressing the current lack of validated companion diagnostics and structured frameworks to guide the integration of these tools into personalized patient care (Figure 1).

Major challenges in DKD management arise from the limitations of current diagnostic approaches. Reliance on albuminuria and estimated glomerular filtration rate (eGFR) is insufficient, as substantial microscopic injury often precedes measurable changes in these indicators (Scilletta et al., 2023; Młynarska et al., 2024). Moreover, a significant proportion of patients exhibit progressive renal decline without prominent albuminuria, resulting in misdiagnosis or delayed detection (Vistisen et al., 2019). Although renal biopsy remains the diagnostic gold standard, its invasiveness, potential complications, and sampling variability limit its routine clinical application (Schnuelle, 2023). These shortcomings often lead to missed opportunities for timely intervention and inadequate risk stratification.

These challenges have catalyzed the search for innovative strategies for early detection, prognosis, and precision treatment that can augment the current standard of care defined by international guidelines such as KDIGO (Kidney Disease: Improving Global Outcomes KDIGO CKD Work Group, 2024). The development of *SGLT2* inhibitors and GLP-1RAs has reshaped the therapeutic landscape, demonstrating robust and glycemia-independent renoprotective benefits (Cai et al., 2025; Tomita et al., 2020). Concurrently, emerging multi-omics platforms are transforming our understanding of DKD pathobiology. Metabolomics has facilitated the discovery of novel circulating biomarkers, while scRNA-seq has illuminated kidney cellular heterogeneity, mapped intercellular communication networks, and uncovered previously unrecognized disease-associated cell states (Wu et al., 2022a; Zhang et al., 2023). In parallel, AI and machine learning (ML) are increasingly applied to integrate electronic health records (EHRs), genomic profiles, and imaging data. These approaches promise enhanced risk prediction, refined patient stratification, and more personalized therapeutic decision-making, ushering DKD research into the era of precision nephrology (Loftus et al., 2022; Meng et al., 2025). This review aims to synthesize these recent transformative advances, with the explicit objective of critically examining the convergence of multi-omics insights, AI-driven analytics, and novel therapeutics, and to outline the pathway for their integration into a new era of precision nephrology for DKD.

## 2 Epidemiology & clinical burden

**FIGURE 2:**
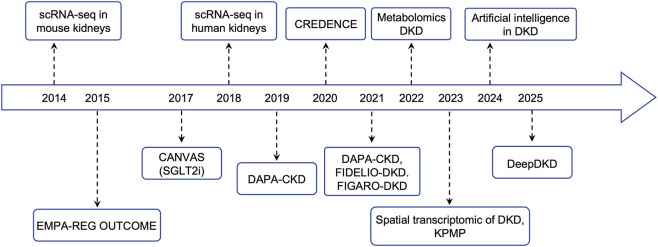
Timeline of key milestones in DKD research. Overview of pivotal advancements that have defined the modern era of DKD research, spanning from foundational discoveries to clinical implementation. The timeline is structured around a central horizontal axis marking the years 2014–2025. Key events are positioned above and below this axis to distinguish between different categories of progress: blue-colored milestones on the upper track primarily represent technological and methodological breakthroughs in molecular profiling and data science, while those on the lower track highlight landmark clinical trials and therapeutic developments.

As the most prevalent microvascular complication of diabetes mellitus, DKD remains the leading cause of chronic kidney disease (CKD) and end-stage kidney disease (ESKD) worldwide (Loftus et al., 2022). Current epidemiological estimates suggest that approximately 30%–40% of individuals with diabetes will develop DKD, thereby markedly elevating the risks of cardiovascular morbidity, disability, and premature death (Balzer et al., 2022; Tan et al., 2024). The global surge in type 2 diabetes, which is largely driven by sedentary behavior, nutritional transitions, and rapid urbanization, has paralleled the rising incidence of DKD, as underscored by recent reports from the International Diabetes Federation (IDF) and the World Health Organization (WHO) (Figure 2) (Pereira et al., 2022; Scilletta et al., 2023; Młynarska et al., 2024).

## 3 Pathobiology & molecular mechanisms of DKD

The pathogenesis of DKD is complex and extends well beyond chronic hyperglycemia. Hemodynamic disturbances, excessive activation of the renin-angiotensin-aldosterone system (RAAS), metabolic derangements, oxidative stress, and notably, persistent low-grade inflammation collectively drive disease initiation and progression (Zhang et al., 2025a; Leoncini et al., 2020). Once considered primarily a structural disorder characterized by glomerulosclerosis, tubular atrophy, and interstitial fibrosis, DKD is now increasingly recognized as a chronic inflammatory disease (Huang et al., 2023). This paradigm shift has been reinforced by evidence of macrophage infiltration and the key roles of pro-inflammatory cytokines and chemokines in perpetuating kidney injury and fibrotic remodeling (Chia et al., 2025; Kang et al., 2025). Genetic susceptibility factors and epigenetic alterations further shape individual vulnerability and the trajectory of renal decline.

## 4 Multi-omics technologies and key discoveries

### 4.1 Single-cell RNA sequencing in DKD

**FIGURE 3:**
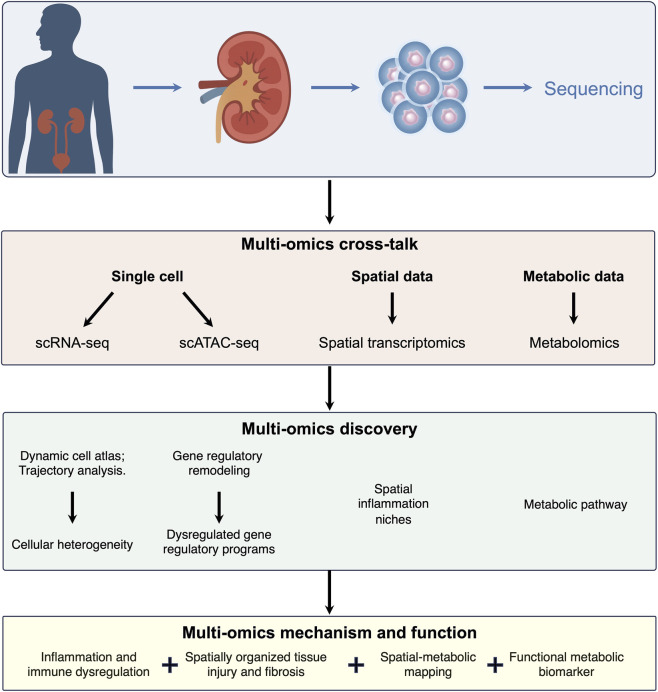
Multi-omics integration framework for DKD research from sample processing to mechanistic insights. The four-tiered flowchart illustrates a comprehensive research pipeline for investigating DKD pathogenesis through integrated multi-omics approaches. The schematic progresses vertically from top to bottom, representing the sequential stages from biological sample collection to mechanistic understanding and functional validation.

The advent of scRNA-seq has paved the way for novel insights into renal pathophysiology, particularly in elucidating the complex cellular dynamics underlying DKD, a condition defined by its profound tissue remodeling (Figure 3). Unlike bulk transcriptomic methods that average gene expression across large cell populations, scRNA-seq dissects the kidney into its smallest functional units, enabling each cell to be profiled individually (Ma et al., 2025a). This fine-grained resolution has dramatically advanced our understanding of how metabolic stress, inflammation, and microvascular injury reshape the diabetic kidney. As comprehensively reviewed by Tan et al., scRNA-seq has become an indispensable tool for annotating cell types, identifying novel subtypes, deciphering intercellular communication, and reconstructing cell differentiation trajectories in DKD (Tan et al., 2024). This methodological framework has been pivotal in shifting research from simple cataloging to the functional dissection of disease mechanisms.

Early investigations using single-cell technologies were mainly devoted to outlining the principal cell types in the kidney, including podocytes, tubular epithelial cells, mesangial cells, vascular endothelial cells and various infiltrating immune populations (Liu et al., 2023). As the number and depth of datasets increased, research attention gradually moved from simple cataloging to exploring the diversity of cell states and the capacity of renal cells to shift between these states under stress. A prominent example is the proximal tubule, which was traditionally regarded as a uniform epithelial segment. Single-cell analyses have now demonstrated that this compartment actually contains several transcriptionally distinct subsets (Zambrano et al., 2022). These subsets exhibit differential sensitivity to high glucose, oxidative injury and excessive protein load, revealing previously unappreciated complexity within this major nephron segment (Lu et al., 2022).

This heterogeneity is critically linked to disease outcomes. A landmark study by Balzer et al. demonstrated that the proximal tubule’s response to injury can diverge into either adaptive repair or a maladaptive, pro-fibrotic state (Balzer et al., 2022). Using a titrated ischemic injury model and scRNA-seq profiling of over 110,000 cells, they identified a distinct maladaptive proximal tubule cell cluster that emerges after severe injury. This cluster is characterized by the expression of proinflammatory and profibrotic cytokines and is associated with vulnerable pathways such as pyroptosis and ferroptosis. Crucially, pharmacological inhibition of these cell death pathways *in vivo* was shown to shift the balance toward adaptive repair and ameliorate fibrosis, directly illustrating the therapeutic potential of targeting specific disease-associated cell states identified through scRNA-seq (Balzer et al., 2022).

### 4.2 Computational trajectory analysis of kidney cells in DKD

Single-cell RNA sequencing has progressively expanded into a diverse range of sophisticated analytical frameworks. Among these, computational trajectory analysis has become a widely adopted method for reconstructing the dynamic evolution of various kidney cell types, including stromal fibroblasts, epithelial cells, and immune populations, throughout the progression of diabetic injury (Tan et al., 2024). Through the application of pseudotemporal ordering tools, researchers have been able to delineate changes in gene expression and cellular fate that unfold during DKD (Fu et al., 2019; Fu et al., 2022). For instance, in glomerular compartments, trajectory inference performed with Monocle successfully ordered endothelial and mesangial cells along a continuum from healthy to diabetic states, revealing gene expression patterns that transition smoothly with disease severity-many of which were not captured by conventional differential expression analysis (Ma et al., 2025b). Similarly, in macrophage populations, Monocle2 uncovered a bifurcated fate trajectory, with each branch defined by a unique gene signature. In the tubular compartment, trajectory analysis using Palantir, applied to integrated single-cell datasets, reconstructed a pathogenic progression starting from injured proximal tubules (iPT) and advancing through urinary aberrant tubular epithelial cells (aTECs) toward fibroblast fates (Zhang et al., 2025b). This trajectory was marked by features of epithelial-to-mesenchymal transition (EMT), providing a mechanistic explanation for both the shedding of degenerative aTECs into the urine and their contribution to fibroblast accumulation in DKD (Ma et al., 2025b). Together, these studies illustrate how trajectory inference can map the continuous molecular and phenotypic transitions that underlie renal pathology.

### 4.3 Integrated multi-omics insights into DKD regulation

Beyond transcriptomic analysis, the integration of single-cell RNA sequencing with assay for transposase-accessible chromatin (scATAC-seq) has enabled deeper insight into how gene regulatory networks are remodeled under diabetic conditions (Figure 3). In one study, integrated scRNA-seq and scATAC-seq profiling of kidneys from db/db mice revealed extensive alterations in both transcriptional output and chromatin accessibility. Key findings included the upregulation of glucose and lipid transporters as well as circadian rhythm-associated transcription factors in proximal tubules, enhanced communication between podocytes and tubular cells, and activation of pathways related to renal injury. These multi-omics findings were further translated to human disease when Wfdc2 was validated as a potential biomarker in DKD patient samples (Shi et al., 2023a). Looking forward, the combination of transcriptomic data with additional proteomic and epigenomic layers will offer an even more powerful strategy for deciphering the regulatory architecture that governs inflammation, tissue remodeling, and fibrogenesis in DKD. Through such integrative approaches, a more comprehensive understanding of DKD pathogenesis is emerging, highlighting novel candidates for diagnostic and therapeutic intervention. Importantly, the true value of multi-omics approaches lies not in individual datasets, but in their integration across molecular layers. Single-cell and spatial transcriptomics define cell-type-specific injury programs and pathogenic microenvironments, whereas metabolomics captures downstream functional consequences at the systemic level. AI further enables the integration of these heterogeneous data types into predictive models that operate at the individual patient level. Together, this layered strategy provides a framework for disease subtyping, early risk stratification, and identification of patients most likely to benefit from targeted interventions.

### 4.4 Immune dysregulation and spatial inflammation in DKD

Integrated analysis of scRNA-seq and spatial transcriptomic data from human DKD and animal models has unequivocally established immune dysregulation as central to disease pathogenesis (Chen et al., 2023). scRNA-seq reveals a significant increase in immune cells, particularly macrophages, which comprise distinct subclusters that dynamically change with progression (Figure 3). Early DKD shows a concomitant rise in both pro-inflammatory and reparative subsets, suggesting an initially balanced response, while late-stage disease is marked by the expansion of specific subsets like the M2-like “M14″macrophage. Increases in T cells, B cells, monocytes, and dendritic cells are also observed, though their roles require further study (Hu et al., 2024). While these foundational atlas studies from Balzer, Susztak, and others have revolutionized our understanding of cellular heterogeneity, it is important to critically acknowledge their limitations. These often include relatively small sample sizes, potential biases introduced by tissue processing for single-cell analysis, and the challenge of fully capturing the spatiotemporal dynamics of disease progression from static snapshots (Balzer et al., 2022; Tan et al., 2024). The integration of spatial transcriptomics is proving essential to validate the *in situ* context of cell states identified in dissociated cells.

Spatial transcriptomics critically resolves the localization of this immune response, showing enrichment of immune cells, especially macrophages, in fibrotic areas (Figure 3). A key finding is the pronounced co-localization of venous endothelial cells and fibroblasts, forming a pro-inflammatory niche. Ligand-receptor analysis deciphers the crosstalk driving inflammation: injured proximal tubular cells adopt a pro-inflammatory phenotype and upregulate chemokines, while fibroblasts significantly overexpress ligands like *CCL2*, *CCL19*, and *CCL21* to recruit immune cells via receptors such as *CCR2* and *CCR7* (Hu et al., 2024). This creates a feed-forward loop of infiltration and inflammation. In summary, DKD progression is driven by a spatially organized process where initial injury triggers chemokine signaling that recruits immune cells, and aberrant stromal-immune crosstalk sustains inflammation and fibrosis, leading to renal decline (Liu et al., 2025).

### 4.5 Metabolomics as an emerging window into DKD biology

DKD remains the most common microvascular complication of diabetes and a major contributor to chronic kidney disease globally. Although eGFR and albuminuria remain central to current diagnostic and prognostic evaluation, these indicators primarily reflect established renal injury rather than early pathogenic changes (Hu et al., 2025). Consequently, there is a critical need for biomarkers capable of capturing the earliest stages of dysfunction, enabling improved risk assessment and providing insight into therapeutic opportunities (Pereira et al., 2022; Van Roy and Speeckaert, 2024). Metabolomics, which systematically characterizes small molecules within biological systems, offers a powerful approach to achieve this aim. Unlike genomics or proteomics, which survey upstream regulatory layers, metabolomics provides an immediate snapshot of biochemical activity and cellular physiology (Kwon et al., 2023).

### 4.6 Analytical advances revealing metabolic disruptions

The rapid evolution of analytical technologies, including mass spectrometry (MS) and nuclear magnetic resonance (NMR) spectroscopy, has accelerated metabolomics-based DKD research (Gurung et al., 2025; Zhang et al., 2025c). Platforms such as GC-MS, LC-MS, and LC-MS/MS enable high-resolution detection of diverse metabolites that reflect mitochondrial activity, amino acid metabolism, lipid turnover, and oxidative stress (Wang et al., 2022; Fan et al., 2024). Altered levels of acyl carnitines and acyl glycines indicate mitochondrial dysfunction, whereas variations in glycine, histidine, and branched-chain amino acids signify disrupted amino acid pathways (Makrecka-Kuka et al., 2017; Pan et al., 2024). Urinary metabolites such as trigonelline and 1-methyl-pyridin-1-ium have shown strong predictive value for accelerated eGFR decline (Valo et al., 2025). Pathway analyses repeatedly highlight abnormalities in glycolysis, lipid handling, tricarboxylic acid cycling, and glutathione-mediated redox regulation (Hasegawa and Inagi, 2021). These findings offer a broad systems-level view of DKD-related biochemical remodeling. Importantly, integration with proteomics and peptidomics significantly enhances interpretability. A large urine-based multi-omics study involving 766 individuals identified distinct metabolites and peptides that varied systematically across DKD stages, with transcriptomic analyses providing robust biological confirmation (Jiang et al., 2023). Machine-learning-supported models using these markers substantially outperformed single-omics approaches, demonstrating powerful diagnostic and staging capabilities (Chen et al., 2024; Li et al., 2025).

### 4.7 Integrative serum omics for noninvasive monitoring

**FIGURE 4:**
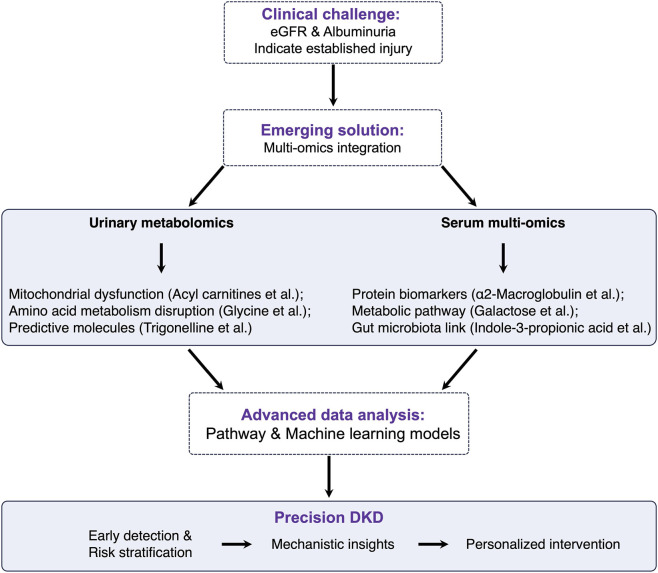
Conceptual workflow for precision management of DKD through multi-omics integration. The figure presents a systematic workflow from clinical challenges to precision solutions. The top box (Clinical challenge) identifies the limitation of conventional biomarkers (eGFR and albuminuria) that only detect established kidney injury. The Emerging solution​ proposes multi-omics integration through two parallel approaches: Urinary metabolomics analyzing mitochondrial dysfunction (acyl carnitines), amino acid metabolism disruption (glycine), and predictive molecules (trigonelline); and Serum multi-omics for comprehensive molecular profiling. These data streams feed into Advanced data analysis using pathway analysis and ML models, ultimately enabling Precision DKD with three key outcomes: early detection and risk stratification, mechanistic insights, and personalized intervention strategies. The visual design employs purple-highlighted headings, light blue boxes, and directional arrows to clearly illustrate the logical progression from problem to solution.

Although multi-omics strategies continue to generate compelling insights, substantial barriers hinder translation into routine care. Validation across diverse and adequately powered cohorts, harmonization of analytical workflows, and development of clinically usable computational tools remain major challenges (Rodriguez-Manzano et al., 2024). Progress is closely tied to advances in bioinformatics, AI, and integrative modeling frameworks. Large initiatives such as the Kidney Precision Medicine Project (KPMP) aim to unify clinical, histological, and multi-omics information, providing a foundation for precision nephrology (de Boer et al., 2021). Given the biological heterogeneity of DKD, relying on a single biomarker is unlikely to achieve sufficient accuracy. Instead, panels combining metabolites, proteins, and peptides that reflect complementary pathophysiological pathways will offer the strongest predictive value (Jiang et al., 2023). Such integrated approaches may eventually support individualized intervention strategies, enabling clinicians to identify high-risk individuals long before irreversible renal injury develops (Figure 4).

### 4.8 Towards an integrated multi-omics framework for clinical translation

While the individual omics layers provide valuable insights, their true potential is unlocked through integration across platforms. A synergistic framework links transcriptomic discoveries of cell-specific injury pathways as spatially resolved by transcriptomic profiling with proteomic or metabolomic validation in accessible biofluids. For instance, a pathogenic process identified by scRNA-seq and localized to specific tissue niches (e.g., fibrotic regions or perivascular areas) through spatial transcriptomics can be tracked non-invasively by measuring corresponding protein or metabolite signatures in serum or urine, bridging mechanistic insight with clinical application. Spatial transcriptomics plays a pivotal role in this integration by providing the missing link between single-cell resolution and tissue architecture, enabling researchers to map pathogenic cell-cell interactions (e.g., immune-stromal crosstalk in fibrotic niches) that drive disease progression and may be reflected in systemic biomarker profiles.

This integrated approach allows for distinguishing core disease drivers from secondary phenomena and facilitates the development of multi-analyte biomarker panels that reflect the multifaceted nature of DKD. Importantly, spatial technologies also help validate whether cell states identified in dissociated single-cell studies truly represent *in situ* biology or may be artifacts of tissue processing. The progression of the field from single-omics discovery towards robust multi-omics verification is exemplified by the growing body of work summarized (Table 1). These studies illustrate the practical application of integrative frameworks, combining various omics layers to uncover novel biology. Furthermore, the translation of these discoveries into clinically viable tools hinges on rigorous validation, a challenge that is systematically addressed (Table 2). This table categorizes promising biomarkers, clearly distinguishing early discovery-phase findings from those that have undergone external replication or are nearing clinical application. Together, these tables provide a consolidated overview of the evidence and the remaining pathway to clinical utility, underscoring that multi-omics integration is essential for generating clinically actionable insights.

**TABLE 1:** Summary of key multi-omics studies in diabetic kidney disease.

Author (Year)	Cohort/Model	Omics layers	Key finding	Level of validation
Hu et al. (2024)	Type 1 diabetic OVE26 mice Human DKD patient biopsies	scRNA-seq Bulk RNA-seq Immunostaining	Diabetic kidneys show dynamic macrophage subset expansion and inflammatory phenotype shifts	scRNA-seq, deconvolution, and immunohistochemical confirmation in human tissues
Shi et al. 2023a)	Mouse model of kidney ischemia-reperfusion injury	scRNA-seq (>110k cells) Bulk RNA-seq	Profibrotic tubules express cytokines; pyroptosis/ferroptosis are targetable	*In vivo* drug validation: pyroptosis inhibition promotes adaptive repair
Tuttle et al. (2022)	37 CKD patients from C-PROBE cohort with 5-year follow-up data	Transcriptomics, proteomics (urine & plasma), metabolomics	Multi-omics revealed 8 urinary proteins and 3 CKD progression pathways	Validated in 94 independent samples
Yaribeygi et al. (2021)	1,146 DKD patients from two EHR-linked biobanks	EHR data, plasma biomarkers	KidneyIntelX ML model predicted DKD progression better than clinical models	Validated in independent cohort (n = 460) with AUC 0.77
Nauck et al. (2021)	Penn Medicine Biobank (n = 573)	Plasma biomarkers, clinical data	KidneyIntelX.dkd enhances DKD progression risk stratification	BioMe (n = 657) CANVAS trial (n = 1197)
Valo et al. (2025)	235 participants	Targeted metabolomics	Urinary succinate independently associated with DKD and albuminuria	Retrospective eGFR correlation analysis
Zambrano et al. (2022)	734,084 retinal images	Retinal imaging AI	AI detects DKD and distinguishes diabetic nephropathy via retinal imaging	65,406 participants across 10 multi-ethnic datasets (AUC 0.791–0.826)

**TABLE 2:** Candidate biomarkers with validation status in diabetic kidney disease.

Author (Year)	Biomarker	Omics source	Discovery cohort	External replication	Clinical assay available
Scholtes et al. (2022)	Model 1: 20-feature signature distinguishing DM vs. non-DM Model 2: 10-feature signature predicting high renal impairment risk in DM patients (includes SNP genes: RPTOR, CLPTM1L, ALDH1L1, LY6D, PCDH9, B3GNTL1, CDS1, ADCYAP, FAM53A) Model 3: 25-feature signature predicting high CKD risk in non-DM patients	Clinical data Untargeted metabolomics Targeted lipidomics Genome-wide SNP	Total n = 618 (Training: n = 557, Testing: n = 61)	Yes	Not mentioned
Yamazaki et al. (2021)	Soluble TNFR-1 Soluble TNFR-2 Kidney injury molecule-1 (KIM-1) Clinical variables (eGFR, UACR, etc.)	Plasma biomarkers Clinical data	CANVAS trial participants with DKD (n = 1,325)	Yes	Likely (KidneyIntelX is a commercially available test from Renalytix AI)
Yaribeygi et al. (2021)	Electronic health record (EHR) data (eGFR, uACR, etc.)	Plasma biomarkers EHR clinical data	Derivation cohort: n = 686 from two EHR-linked biobanks	Yes	Likely (KidneyIntelX is a commercially available test from Renalytix AI)
Van Roy and Speeckaert. (2024)	Wfdc2	scRNA-seq scATAC-seq	db/db diabetic mice Human DKD patients	No	No
Pan et al. (2024)	Myo-inositol Choline Citrate Mannose Other monosaccharides and TCA cycle metabolites	Targeted metabolomics using nuclear magnetic resonance (NMR)	DKD patients (n = 208, stages 1–5) Healthy controls (n = 26)	No	Not mentioned
Wu et al. (2022b)	Retinal imaging features	Retinal fundus photography Deep learning algorithm	Singapore Integrated Diabetic Retinopathy Program (SiDRP, n = 6,066)	Yes (4 external validation cohorts) 1. SEED study (Singapore, n = 1885) 2. SMART2D (Singapore, n = 439) 3. AHES (Australia, n = 460) 4. NICOLA (Northern Ireland, n = 265)	Not mentioned
Jiang et al. (2020)	Taurine and hypotaurine metabolism Tryptophan metabolism Tyrosine metabolism	Label-free quantification proteomics Non-targeted metabolomics	DKD mouse model	No	Not mentioned

## 5 Artificial intelligence & machine-learning in DKD

### 5.1 A retinal imaging breakthrough for DKD

The application of AI in DKD care has reached a significant milestone with the development of highly specialized systems like DeepDKD (Meng et al., 2025). This deep learning model directly addresses two critical clinical challenges: the accessibility of screening and the complex differentiation of isolated diabetic nephropathy from non-diabetic kidney disease (NDKD) (Liang et al., 2013). DeepDKD’s innovation lies in its use of retinal fundus images as a non-invasive diagnostic tool, leveraging the well-established connection between microvascular damage in the eye and the kidney (Navaneethan et al., 2023; Wong et al., 2018). Its development followed a rigorous, population-based, multi-stage process. For DKD detection, the model was pretrained on a vast dataset of over 734,084 images. Subsequent internal validation on a cohort of 121,578 participants demonstrated high accuracy, with an area under the receiver operating characteristic curve (AUC) of 0.842 (Meng et al., 2025). More importantly, its robustness was confirmed through extensive external validation across ten multi-ethnic datasets from five countries, where it maintained strong performance (AUCs 0.791–0.826). For the more nuanced task of differentiating types of kidney disease, DeepDKD was trained and tested on data from patients who had undergone kidney biopsies. It achieved an impressive AUC of 0.906 internally and AUCs of 0.733–0.844 externally. The clinical utility was further solidified through proof-of-concept studies. In a prospective primary care study, DeepDKD significantly outperformed a standard clinical metadata model (sensitivity: 89.8% vs. 66.3%). Longitudinal validation over 4.6 years confirmed its clinical utility: patients classified by DeepDKD as having isolated diabetic nephropathy showed slower eGFR decline (27.45%) compared to those classified as NDKD (52.56%) (Meng et al., 2025). This long-term risk stratification capability, unattainable with conventional models, demonstrates DeepDKD’s potential as an accessible screening tool in resource-limited settings. Importantly, it may refine risk assessment beyond current Kidney Disease: Improving Global Outcomes (KDIGO) guideline parameters, and reduce reliance on invasive biopsies (Kidney Disease: Improving Global Outcomes KDIGO CKD Work Group, 2024).

### 5.2 Robust performance and persistent challenges

The remarkable performance of DeepDKD is not an isolated phenomenon but is instead reflective of the broader, high-performing potential of ML in DKD risk prediction, as confirmed by a recent systematic review and meta-analysis (Li et al., 2025; Makino et al., 2019; Betzler et al., 2023). This analysis, synthesizing evidence from 26 studies, found that ML models demonstrate robust and excellent discriminatory ability for predicting DKD development and progression in patients with type 2 diabetes (Makino et al., 2019). The pooled AUC was 0.839 in internal validation sets and a nearly identical 0.830 in external validation sets, indicating strong generalizability across independent patient populations. Subgroup analyses provide critical insights into the relative strengths of different algorithmic approaches. Deep learning models, like the convolutional neural network underpinning DeepDKD, achieved the highest pooled AUC of 0.863, outperforming both other ML models (pooled AUC 0.811) and traditional regression-based techniques like logistic regression (AUC 0.797). Among specific algorithms, random forest led with an AUC of 0.848 (Meng et al., 2025). The review also highlighted the diverse and complex data types that fuel these models, which extend far beyond basic clinical variables like demographics, HbA1c, and blood pressure (Ceriello et al., 2017; Jiang et al., 2020). Successful models have incorporated advanced data, including circulating metabolites, genetic attributes, histopathological results, retinal photographs, and radiomic signatures, showcasing ML’s unique capacity to integrate multidimensional information. However, this promising landscape is tempered by significant methodological challenges that currently hinder widespread clinical adoption. A critical finding was a high risk of bias in most studies, primarily due to issues in statistical analysis such as inadequate calibration assessment, improper handling of missing data, and insufficient internal validation. Furthermore, external validation was performed in only eight of the 26 studies, and methodologies for handling data heterogeneity were inconsistent.

### 5.3 Translational challenges and future directions for AI in DKD

The promising performance of AI models must be tempered by a clear-eyed assessment of the barriers to their clinical implementation. A prime example that illustrates both the potential and the pathway is the KidneyIntelX risk score. Developed using a machine-learning algorithm on data from EHR-linked biobanks, KidneyIntelX integrates plasma biomarkers with clinical variables to predict DKD progression (Coca and Nadkarni, 2025). Its development and validation journey highlights several key considerations raised by the reviewer. First, regarding external validation and generalizability, KidneyIntelX has been successfully validated in independent, multi-ethnic cohorts such as the BioMe Biobank and the multinational CANVAS clinical trial population, demonstrating robust performance (AUC ∼0.77) superior to standard clinical models and KDIGO risk categories (Chan et al., 2021; Nadkarni et al., 2023). Furthermore, analyses from CANVAS suggested that individuals stratified as high-risk by KidneyIntelX may derive greater renal benefit from *SGLT2* inhibitor treatment, indicating its potential for therapy personalization (Nadkarni et al., 2022; Lam et al., 2022).

However, the KidneyIntelX case also underscores persistent challenges. Bias related to ancestry and healthcare systems remains a concern, as even validated models can underperform in populations not represented in training data, necessitating ongoing validation in diverse global cohorts. Model interpretability, or the “black box” problem, is another hurdle; while complex models like random forests can achieve high accuracy, providing clinicians with understandable reasons for a risk prediction is crucial for trust and adoption. Efforts in explainable AI (XAI) are essential to address this. Finally, regulatory and implementation challenges are significant. KidneyIntelX’s journey toward FDA recognition exemplifies the rigorous pathway required to translate an algorithmic risk score into a clinically deployed test, involving analytical validation, clinical validation, and demonstration of clinical utility (Coca and Nadkarni, 2025).

### 5.4 A transformative shift in disease management

**FIGURE 5:**
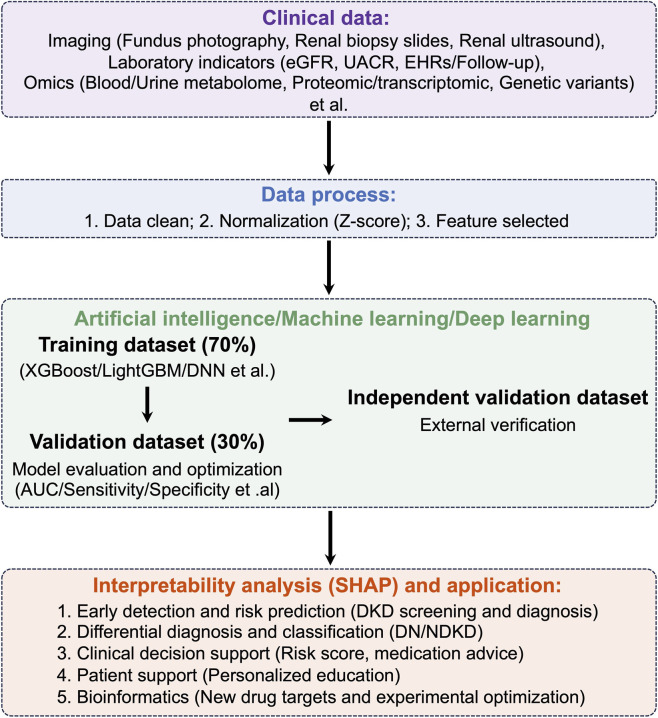
AI-driven workflow for DKD analysis using clinical and omics data. The figure presents a vertical workflow for AI-based DKD analysis. Clinical data collection (purple box) includes imaging (fundus photography, renal biopsy, ultrasound), laboratory indicators (eGFR, UACR, EHRs), and omics data (metabolome, proteome, transcriptome, genetics). Data processing (blue box) involves cleaning, Z-score normalization, and feature selection. Processed data is split into training (70%) for model development (XGBoost, LightGBM, DNN) and validation (30%) for evaluation (AUC, sensitivity, specificity), with an independent validation set for external verification. Interpretability analysis (SHAP) and applications (red box) include: (1) early detection/risk prediction, (2) differential diagnosis (DN vs. NDKD), (3) clinical decision support, (4) patient education, and (5) bioinformatics research. Color-coded boxes connected by downward arrows clearly show the workflow from data to clinical implementation.

Beyond the specific predictive models validated in research settings, the application of AI and ML is catalyzing a transformative shift across the entire spectrum of DKD management (Figure 5). The field has evolved from early studies using basic algorithms to identify clinical indicators to highly sophisticated approaches that leverage large-scale EHRs and complex data structures. For instance, models using gradient-boosted trees on data from hundreds of healthcare sites have been shown to outperform traditional risk scores. This predictive power is being refined through the incorporation of longitudinal and even unstructured EHR data to capture dynamic disease patterns, and by focusing on specific subgroups, such as patients with normoalbuminuria, to challenge existing clinical paradigms. The utility of AI extends beyond simple prediction into several key areas (Shi et al., 2023b). In risk quantification, integrated platforms like the FDA-recognized KidneyIntelX, which combines biomarkers with EHR data, have proven superior to traditional models like KDIGO, offering improved precision for personalized care (Chan et al., 2021; Lam et al., 2022). In early detection, AI enables innovative non-invasive methodss; for example, deep learning models applied to standard 12-lead electrocardiograms have shown high accuracy in identifying renal impairment, complementing the approach taken by retinal image-based systems like DeepDKD. Furthermore, ML is powerful in biomarker discovery, where techniques like non-targeted metabolomics analyzed with deep learning have uncovered a wider spectrum of potential biomarkers for kidney function decline than conventional methods (Betzler et al., 2023; Chan et al., 2021; Basuli et al., 2025). This study exemplifies the power of AI-driven multi-omics integration, developing a model that combines clinical data, metabolomics, lipidomics, and genomics to identify a biomarker signature for predicting DKD risk with high accuracy (AUC up to 0.89), while also revealing potential underlying molecular networks (Wu et al., 2022b). AI is even being applied to novel drug target identification, using innovative algorithms to predict new therapeutic avenues (Loftus et al., 2022). These research advancements are rapidly translating into tangible clinical tools, with commercial platforms from companies like pulseData and Google Health emerging for predicting kidney failure and monitoring patient health (Basuli et al., 2025; Datar et al., 2021). This collective progress, encompassing predictive modeling, risk stratification, non-invasive screening, and biomarker discovery, signals a definitive move towards a more proactive, personalized, and effective strategy for preventing and managing DKD, with the ultimate goal of improving patient outcomes through earlier and more precise interventions.

## 6 Translational biomarkers & diagnostic platforms (blood, urine, imaging)

Metabolomic signatures face significant challenges due to confounding by extrinsic factors (diet, medications including SGLT2i/GLP-1RAs, comorbidities) and the predominance of cross-sectional studies with modest sample sizes, limiting causal inference and generalizability. While numerous discovery-phase metabolites show promise, few have demonstrated incremental value over standard parameters (eGFR, UACR) in large prospective cohorts. This underscores the need for longitudinal multicenter studies and standardized analytical pipelines to enable clinical translation.

Despite these challenges, serum-based multi-omics approaches offer a promising minimally invasive strategy. A large multicenter study (n = 1,513) demonstrated that integrating proteomic (alpha-2-macroglobulin, cathepsin D, CD324) and metabolomic (galactose, glycerolipid pathways, glycerol-3-galactoside) data substantially improved classification precision versus single-omics approaches (Liu et al., 2021). This integration provides a valuable resource for biomarker development, though further independent validation remains essential. Multi-omics also reveals the gut microbiota’s role, reduced indole-3-propionic acid (a microbiota-derived metabolite) associates with DKD, and supplementation preserves SIRT1 and mitochondrial function (Li et al., 2022). Similarly, nutritional interventions like anthocyanins modulate taurine, hypotaurine, tryptophan, and tyrosine metabolism, highlighting diet-microbiome-metabolism interactions as therapeutic targets (Zeng et al., 2025). These findings illustrate the potential of integrated multi-omics approaches to bridge mechanistic discovery with clinical surveillance while acknowledging current limitations.

Deep learning models like DeepDKD represent a breakthrough in non-invasive DKD screening by analyzing retinal fundus images, leveraging the retino-renal microvascular connection (Meng et al., 2025). Pretrained on 734,084 images, DeepDKD achieved high accuracy (AUC 0.842) in internal validation (n = 121,578) and maintained robust performance (AUCs 0.791–0.826) across ten multi-ethnic external datasets. For distinguishing diabetic nephropathy from non-diabetic kidney disease, it attained AUCs of 0.906 internally and 0.733–0.844 externally in biopsy-confirmed cases. Prospective validation demonstrated superior sensitivity (89.8%) versus clinical models, with longitudinal data showing accurate risk stratification (27.45% vs. 52.56% eGFR decline in classified groups over 4.6 years). This AI-powered imaging platform offers accessible, non-invasive screening with potential to supplement or reduce reliance on invasive biopsies.

## 7 Therapeutic landscape & precision therapeutics

### 7.1 Established therapeutic pillars and their mechanistic foundations

**FIGURE 6:**
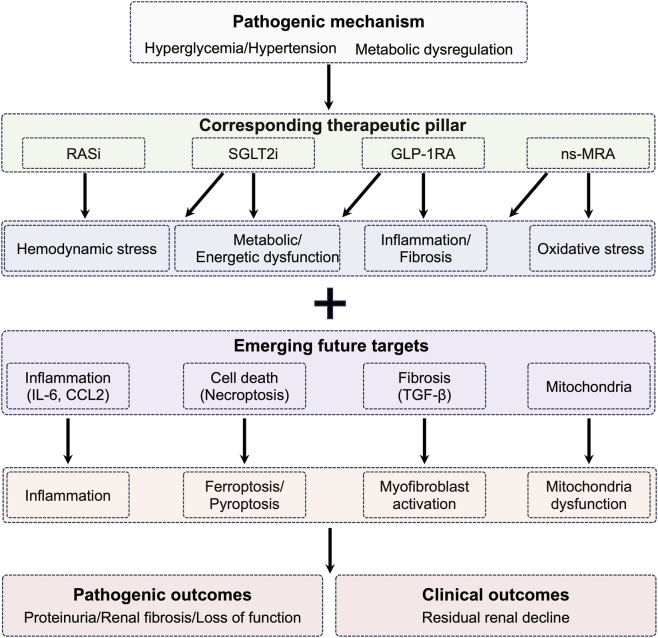
Pathogenic mechanisms, therapeutic strategies, and clinical outcomes in DKD. The figure illustrates the interplay between pathogenic mechanisms (hyperglycemia/hypertension and metabolic dysregulation) leading to hemodynamic stress, metabolic dysfunction, inflammation/fibrosis, and oxidative stress. Current therapeutic pillars (RASi, SGLT2i, GLP-1RA, ns-MRA) target these pathways, while emerging future therapies focus on inflammation (IL-6, CCL2), cell death (necroptosis, ferroptosis, pyroptosis), fibrosis (TGF-β, myofibroblast activation), and mitochondrial dysfunction. Pathogenic outcomes include proteinuria, renal fibrosis, and loss of kidney function, ultimately resulting in clinical outcomes of residual renal decline. The diagram uses a clear black-and-white layout with boxes and arrows to show causal relationships from mechanisms through interventions to outcomes.

The management of DKD has undergone a substantial transformation with the emergence of four cornerstone pharmacological classes that consistently attenuate renal decline: inhibitors of the renin-angiotensin system (RAS) (Yaribeygi et al., 2021; Nauck et al., 2021), *SGLT2* inhibitors (Vallon and Verma, 2021; Yamazaki et al., 2021), GLP-1RAs (Marso et al., 2016; Mann et al., 2017), and non-steroidal mineralocorticoid receptor antagonists (ns-MRAs) such as finerenone (Scholtes et al., 2022; van Raa et al., 2024) (Figure 6). Evidence from pivotal trials, including EMPA-KIDNEY (Fernández-Fernandez et al., 2023; The EMPA-KIDNEY Collaborative Group et al., 2023), DAPA-CKD (Heerspink et al., 2020; Fletcher et al., 2023), CREDENCE (Oshima et al., 2020; Verma et al., 2020), FIDELIO-DKD (Agarwal et al., 2022; Sato and Nishimoto, 2025), FLOW (Bae, 2025), and Clinical Phenotyping and Resource Biobank Core (C-PROBE) (Alakwaa et al., 2025), has firmly established their efficacy, leading to their incorporation into the contemporary standard of care as outlined in the KDIGO clinical practice guidelines (Kidney Disease: Improving Global Outcomes KDIGO CKD Work Group, 2024; Kidney Disease: Improving Global Outcomes KDIGO Diabetes Work Group, 2022). The DAPA-CKD trial, for instance, demonstrated that dapagliflozin significantly reduced the risk of a composite outcome of sustained eGFR decline, end-stage kidney disease, or renal/cardiovascular death by 39% in patients with chronic kidney disease, with consistent benefits observed in participants with and without type 2 diabetes (Heerspink et al., 2020). These agents confer kidney protection through diverse biological actions that extend beyond blood pressure reduction or glycemic improvement. Their benefits encompass correcting glomerular hemodynamic imbalance, reducing podocyte stress, alleviating tubulointerstitial inflammatory responses, and suppressing fibrotic remodeling (Savedchuk et al., 2023). For example, *SGLT2* inhibition restores tubuloglomerular feedback and improves renal metabolic efficiency (Upadhyay, 2024), whereas GLP-1RAs and finerenone demonstrate pronounced anti-inflammatory and anti-fibrotic activities (Tran et al., 2024; Alharbi, 2024). Current clinical practice increasingly supports a layered treatment paradigm, often combining RAS blockade with *SGLT2* inhibition and, when appropriate (van Raa et al., 2024), ns-MRAs or GLP-1RAs according to the patient’s cardiovascular and renal risk burden. Despite these advances, real-world implementation remains suboptimal due to economic barriers, therapeutic inertia, and limited awareness in frontline care settings (Stougaa et al., 2025).

### 7.2 Emerging pathogenic mechanisms and opportunities for innovative therapies

Although these established therapies substantially slow disease progression, many individuals with DKD continue to experience renal deterioration, prompting intensified investigation into novel pathogenic processes. Several pathways have recently gained attention (Zhao et al., 2025; Tuttle et al., 2022; Ghose et al., 2024). Dysregulated angiogenesis, particularly through angiopoietin-2-driven endothelial dysfunction, appears to contribute to microvascular instability (Zhao et al., 2025; Wang et al., 2021). Mitochondrial impairment is another central factor (Fan et al., 2024; Ahmad et al., 2021), with the release of mitochondrial DNA activating innate immune sensors such as the cGAS-STING axis and promoting regulated cell-death programs including ferroptosis, necroptosis, and pyroptosis (Shen et al., 2022; Huang et al., 2025). Persistent low-grade inflammation mediated by cytokines, such as TNF, IL-6, and CCL2, further accelerates tissue damage (Zhou et al., 2025a). Complement activation, evidenced by renal deposition of C3 and membrane attack complex components, is increasingly recognized as pathogenic. Additional promising targets include TGF-β-driven fibrogenic signaling and oxidative stress pathways propagated by advanced glycation end-products and their receptor RAGE (Rani et al., 2025). Multiple early-phase clinical programs have evaluated agents modulating these mechanisms, such as CCR2/CCL2 blockade (emapaticap pegol, CCX140-B) (Zhou et al., 2025b), IL-6 neutralization (ziltivekimab), and anti-fibrotic candidates like pirfenidone or ASK1 inhibition (selonsertib) (Badal et al., 2022; Petrovic et al., 2025) (Figure 6). Although clinical outcomes have varied, with some showing reductions in albuminuria and others failing to meet primary endpoints, their collective results reinforce the biological relevance of these pathways and justify continued therapeutic exploration. Other interventions, such as fenofibrate’s enhancement of fatty-acid oxidation, have shown renal benefit in observational research and warrant further assessment.

### 7.3 Bridging mechanistic discovery and therapeutic testing: the role of omics and AI

The translation of these emerging therapeutic targets into proven therapies requires a new paradigm for clinical trial design. Here, multi-omics and AI transition from being discovery tools to becoming enablers of precision medicine. Rather than testing novel agents in broad, heterogeneous DKD populations, these technologies allow for precision trial enrichment. This involves using omics-derived signatures or AI-driven risk profiles to identify patient subgroups that are most likely to respond to a specific mechanism-based therapy. This approach increases the statistical power and efficiency of clinical trials by enriching for a population with a higher anticipated effect size, thereby reducing trial size, cost, and duration. Ultimately, successful biomarkers used for enrichment could evolve into companion diagnosticsto guide therapeutic decisions in clinical practice. This framework moves the discussion beyond conceptual promise and outlines a concrete pathway through which the biological insights detailed in this review can be rigorously tested and translated into personalized patient care.

## 8 Challenges, gaps & opportunities for clinical translation

Metabolomic profiling captures both endogenous metabolites and exogenous compounds, elucidating how genetic, environmental, and metabolic factors shape disease states. Its sensitivity to subtle biochemical changes makes it promising for early DKD detection and metabolic pathway delineation (Jung et al., 2024). However, critical limitations persist, metabolomic signatures are confounded by diet, medications, and comorbidities, while most studies remain cross-sectional with modest sample sizes, limiting causal inference and clinical translation.

Similarly, AI models face substantial translational challenges. Many are trained on retrospective datasets susceptible to hidden biases (population structure, imaging devices, healthcare access), and struggle with interpretability and calibration across diverse settings. The incremental benefit beyond established risk markers must be rigorously demonstrated in prospective studies before widespread adoption (Dholariya et al., 2024). These barriers—shared by both metabolomics and AI approaches, highlight the need for methodological rigor, data transparency, and robust multicenter validation to ensure safety, efficacy, and generalizability.

Ultimately, while these technologies represent breakthroughs in DKD risk prediction and early detection, their clinical success hinges on overcoming challenges related to generalizability, bias, interpretability, and regulatory approval. Future efforts must prioritize developing fair, transparent, and clinically integrated tools that not only predict risk but demonstrably improve patient outcomes.

## 9 Roadmap & research priorities (short-term and long-term)

We propose the following prioritized roadmap to guide future progress: (1) Prioritize multi-omics data standardization and integration. Establish standardized protocols for data generation across platforms and centers, alongside robust, open-source computational frameworks, to enable the construction of large-scale, integrated multi-omics reference maps of DKD progression. (2) Establish large, diverse, and deeply phenotyped longitudinal cohorts. Move beyond cross-sectional designs by developing multi-ethnic cohorts with longitudinal biospecimen collection and comprehensive clinical annotation, enabling rigorous validation of biomarkers and AI models across populations and disease stages. (3) Develop and validate clinically actionable AI-driven tools. Focus on interpretability, fairness, and prospective real-world validation to ensure that AI-based models provide incremental value over existing standard-of-care risk stratification approaches. (4) Conduct biomarker-enriched, mechanism-based clinical trials. Leverage molecular signatures to enrich for patient subgroups most likely to respond to targeted therapies, thereby increasing trial efficiency and improving the likelihood of therapeutic success. (5) Address regulatory and health economic challenges. Engage proactively with regulatory agencies to define approval pathways for multi-omics and AI-based diagnostics, while conducting health economic analyses to demonstrate clinical utility and cost-effectiveness. (6) Create interdisciplinary training and data-sharing ecosystems. Foster sustained collaboration among nephrologists, computational scientists, and clinical trialists, supported by secure and interoperable data-sharing platforms.

Short-term research priorities (3–5 years): Establish and validate integrated multi-omics-AI diagnostic models; validate the clinical utility of lead biomarkers (e.g., specific metabolites, proteins, and radiomic features) in large prospective cohorts; advance precision therapeutic trials targeting validated pathways (e.g., inflammation, fibrosis) and explore their combination with SGLT2i/GLP-1RAs; develop interpretable, bias-resistant AI clinical decision support prototypes.

Long-term research priorities (5–10 years): Achieve real-time dynamic integration of multi-omics data with electronic health records to build personalized digital twin models for risk prediction and treatment simulation; complete phase III trials of novel therapies (e.g., targeting mitochondrial function, cellular senescence, microbiome) and facilitate their clinical translation; establish an AI-driven, multimodal biomarker-guided precision paradigm for DKD prevention, diagnosis, and treatment, and complete its health economic evaluation.

## 10 Conclusion

Recent advances in single-cell and metabolomic technologies have profoundly expanded our understanding of DKD by uncovering previously unrecognized cellular states, pathogenic microenvironments, and metabolic disturbances. Single-cell and spatial profiling identify maladaptive tubular transitions, inflammatory niches, and endothelial dysfunction as central drivers, while metabolomics reveals complementary disruptions in amino acid utilization, mitochondrial pathways, and lipid metabolism. Together, these approaches provide an integrated, multi-layered view of DKD pathogenesis, enabling earlier identification of high-risk individuals.

In parallel, AI and emerging therapeutics are accelerating clinical translation. Deep learning models integrating imaging, multi-omics, and EHR data enhance early detection and risk prediction, while novel pharmacologic strategies now target inflammation, mitochondrial injury, fibrosis, and complement activation beyond traditional targets (Loftus et al., 2022). The convergence of omics-derived biomarkers, AI-driven decision support, and mechanism-based drug development is fundamentally reshaping the therapeutic landscape. Precision medicine in DKD builds upon established frameworks like KDIGO (Kidney Disease: Improving Global Outcomes KDIGO CKD Work Group, 2024), transitioning from population-based algorithms to individualized care. AI-assisted risk stratification using non-invasive imaging, serum multi-omics panels, and layered pharmacotherapy (e.g., SGLT2i with ns-MRAs) appears particularly poised for near-term clinical translation.

Despite this progress, realizing precision nephrology’s full potential requires coordinated efforts to overcome translational barriers in data integration, clinical validation, and implementation. By systematically addressing these priorities, the field can transition from reactive, one-size-fits-all management toward proactive, personalized precision care, ultimately improving long-term patient outcomes.

## 11 Literature search strategy

A systematic literature search was conducted in the PubMed database to identify relevant publications. The search spanned articles published from January 2015 to December 2025, employing a combination of Medical Subject Headings (MeSH) and key terms, including: ‘diabetic kidney disease’, ‘multi-omics’, ‘single-cell RNA sequencing’, ‘metabolomics’, and ‘artificial intelligence’. The initial search yielded 47,036 records. To ensure the inclusion of current evidence, 26,731 records published more than 10 years ago were excluded prior to screening, resulting in 20,305 records for title and abstract assessment.

Following the removal of 835 irrelevant records based on abstract screening, the full texts of 19,470 reports were sought for retrieval. Eligibility for inclusion was strictly restricted to the following publication types to maintain a focus on high-level evidence: Classical Article, Clinical Study (encompassing Clinical Trial and Randomized Controlled Trial), Meta-Analysis, and Review. Application of this filter resulted in 14,033 reports. Subsequently, 5,437 reports were formally assessed for eligibility based on full-text review. Of these, 5,399 reports were excluded for the following reasons: 4,495 did not substantively address the core multi-omics or artificial intelligence keywords, and 904 were excluded as their article type did not provide associated research data. Ultimately, 38 studies met all pre-defined criteria and were included in the final review. This review is reported according to the Preferred Reporting Items for Systematic Reviews and Meta-Analyses (PRISMA) statement 2020 (Page et al., 2021). The study selection process is detailed in the PRISMA flow diagram (Supplementary Figure S1).

## Supplementary material

SUPPLEMENTARY FIGURE S1 PRISMA 2020 flow diagram for systematic review of diabetic kidney disease incorporating multi-omics and artificial intelligence applications. The diagram illustrates the study selection process, from initial identification to final inclusion. A total of 47,036 records were identified through a systematic search of PubMed. After excluding 26,731 records published more than 10 years ago, 20,305 records underwent title and abstract screening, with 835 excluded as irrelevant. Full-text retrieval was attempted for 19,470 reports. Eligibility was restricted to Classical Articles, Clinical Studies (including Clinical Trials and Randomized Controlled Trials), Meta-Analyses, and Reviews, yielding 14,033 reports. Following full-text assessment of 5,437 reports, 5,399 were excluded (4,495 for not addressing core keywords and 904 for lacking associated research data). Ultimately, 38 studies met all inclusion criteria and were included in the review.
