# Supplementary figures and images for: Diabetic kidney disease: integrating multi-omics insights, artificial intelligence, and novel therapeutics for precision medicine
Source: Front Genet. 2026 Jan 20;17:1760654. doi: 10.3389/fgene.2026.1760654 (PMC12863704; doi:10.3389/fgene.2026.1760654)

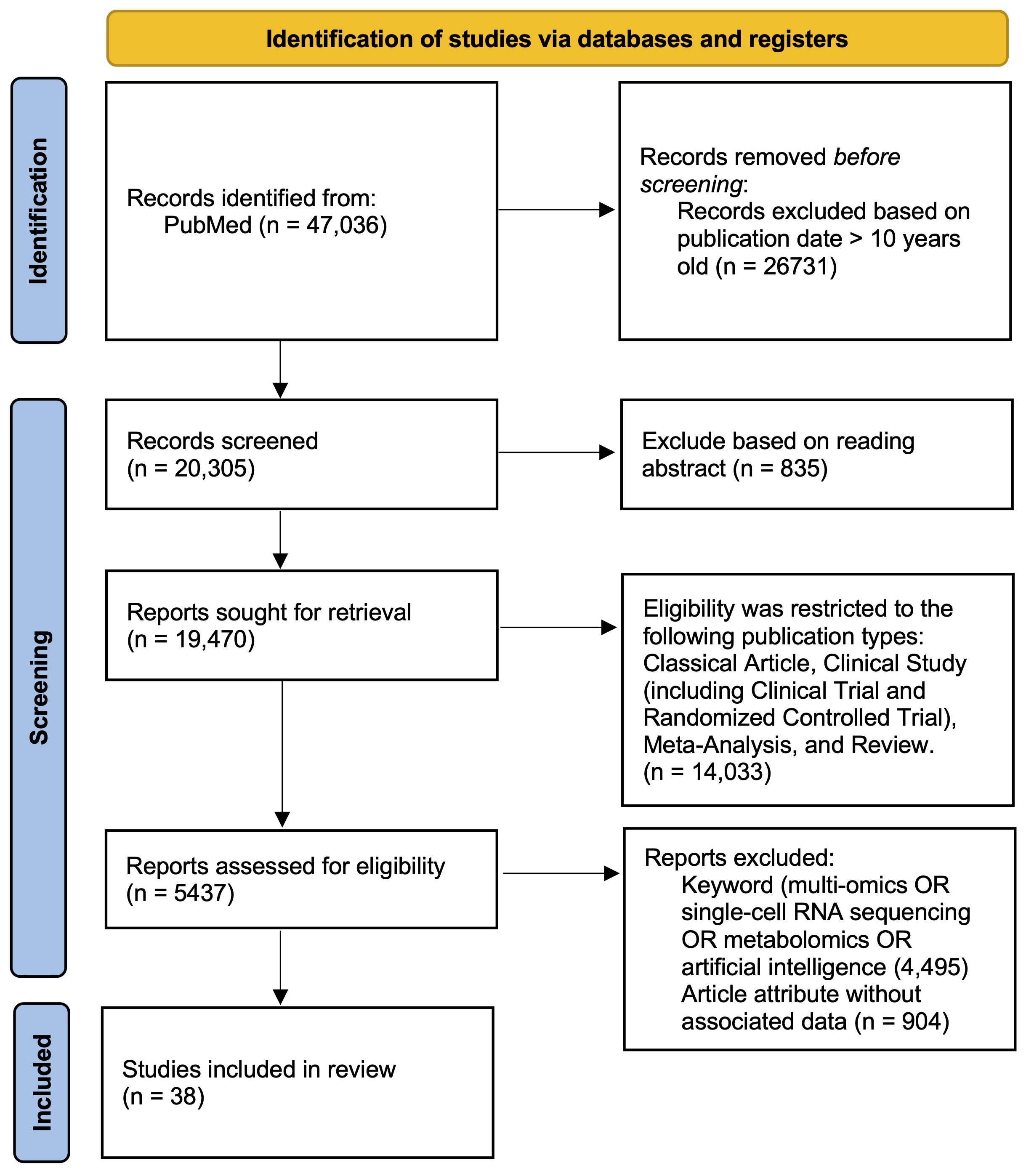

Supplement: Supplementary file 1 [file Image1.jpeg]
